# MedFuseNet: An attention-based multimodal deep learning model for visual question answering in the medical domain

**DOI:** 10.1038/s41598-021-98390-1

**Published:** 2021-10-06

**Authors:** Dhruv Sharma, Sanjay Purushotham, Chandan K. Reddy

**Affiliations:** 1 Department of Computer Science, Virginia Tech, Arlington, VA USA; 2 Department of Information Systems, University of Maryland Baltimore County, Maryland, USA

**Keywords:** Computer science, Medical imaging

## Abstract

Medical images are difficult to comprehend for a person without expertise. The scarcity of medical practitioners across the globe often face the issue of physical and mental fatigue due to the high number of cases, inducing human errors during the diagnosis. In such scenarios, having an additional opinion can be helpful in boosting the confidence of the decision maker. Thus, it becomes crucial to have a reliable visual question answering (VQA) system to provide a ‘second opinion’ on medical cases. However, most of the VQA systems that work today cater to real-world problems and are not specifically tailored for handling medical images. Moreover, the VQA system for medical images needs to consider a limited amount of training data available in this domain. In this paper, we develop *MedFuseNet*, an attention-based multimodal deep learning model, for VQA on medical images taking the associated challenges into account. Our *MedFuseNet* aims at maximizing the learning with minimal complexity by breaking the problem statement into simpler tasks and predicting the answer. We tackle two types of answer prediction—categorization and generation. We conducted an extensive set of quantitative and qualitative analyses to evaluate the performance of *MedFuseNet*. Our experiments demonstrate that *MedFuseNet* outperforms the state-of-the-art VQA methods, and that visualization of the captured attentions showcases the intepretability of our model’s predicted results.

## Introduction

According to World Health Organization (WHO)^1^, over 45% of the countries across the globe have less than one physician available per 1000 population. This burdens each medical practitioner to examine a large number of medical reports, which increases the likelihood of human error due to fatigue^2^. Computer-Aided Diagnosis (CAD) systems^3^ have proven to reduce human-generated medical errors. Moreover, CAD systems can also help provide deeper insights into the case, which might not be comprehensible to a naked eye, and thus are very useful for providing a second opinion to the doctor. The push towards digital delivery of medical reports to patients and doctors via CAD enhanced online portals has resulted in better communication of information to the patients. These portals can provide good interfaces to the patients for reliable and trustworthy information directly from doctors or healthcare providers compared to the vast amount of misleading information available online. Moreover, these portals augmented with automated intelligent systems such as a visual question answering system can help divert a lot of patient communication traffic from hospitals and doctors, thus reducing their stress. The primary focus of this paper is the development of an automated visual question answering system for the medical domain.

The advancements in the field of deep learning have demonstrated tremendous success in achieving state-of-the-art results in various problems in the fields of computer vision, natural language processing, information retrieval, to name a few. This was primarily due to the recent enhancements in the computational power of the machines and the development of new learning and optimization methods for neural networks. Several application domains have also benefited enormously due to these recent advances. In particular, the medical domain has seen a significant boost in the use of deep learning techniques for gathering more meaningful insights about various complex data sources ranging from radiology scans to medical records. Significant improvements in the performance metrics have been recorded for tasks related to image understanding, such as segmentation of tumors present in brain^4^, skin^5^, and other organs^6^. There has also been much compelling research done in natural language processing tasks (NLP) and medical records, such as the predictive analysis using clinical records of patients^7,8^. A more interesting problem is the one with both vision and NLP components—Visual Question Answering (VQA). VQA aims to answer a natural language question associated with an image. In the medical domain, an image corresponds to a radiology scan of a patient accompanied by a clinically relevant question-answer pair, where the answer might belong to a pre-defined limited set or can be a sequence of words.

Apart from being a problem related to both Computer Vision and NLP (i.e., multimodal components), VQA for the medical domain has its own new challenges^9^. The main challenge is the limited availability of labeled medical data due to the patients’ privacy concerns. Moreover, the labeling or annotation of the available medical data is in itself a challenge due to the limited number (and availability) of practitioners/experts. As a result, the number of VQA datasets available in the medical domain and the number of VQA data samples in them are quite less compared to the VQA datasets for the other real-world domains. In fact, the medical VQA datasets have data points in the order of hundreds to a few thousands^10^, while the popular VQA datasets have hundreds of times more data points^11^. Thus, the limited data poses a challenge in using the existing deep learning-based VQA approaches for VQA in the medical domain. As VQA deals with multimodal data inputs (natural language question and an associated image), it is important to maximize the information from these two modalities. In the medical domain, the medical data is implicitly complicated due to the high amount of information packed in a single clinical report or a radiology scan. The scan or report could be for any anatomical region, and there could be noise or artifacts induced during scanning or while documenting clinical reports. Thus, a good VQA system for the medical domain should handle these data availability and heterogeneity challenges. Another challenge for VQA is the generation of the answer i.e., the model should output a meaningful sequence of words, which we refer to as the answer generation task. Furthermore, in the medical domain, the transparency and trustworthiness of the model’s predictions are needed, and therefore, VQA results should be interpretable. Thus, there is a need to develop novel approaches for VQA in the medical domain, which can judiciously use the available limited annotated medical data to minimize the answer prediction and answer generation errors, and at the same time, provide interpretable results.

**Figure 1:**
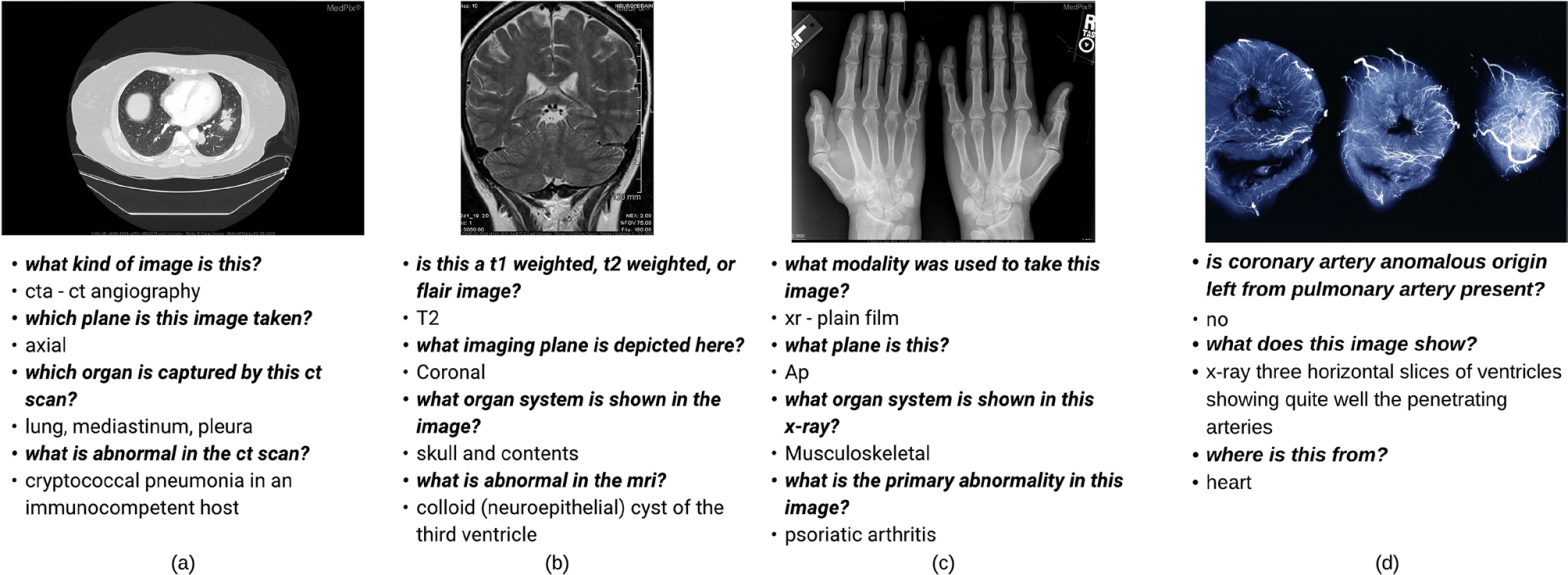
Sample radiology scans and the corresponding question-answer pairs from the MED-VQA and PathVQA dataset. The first three (**a**–**c**) belong to the MED-VQA dataset and the last one (**d**) belongs to the PathVQA dataset.

**Figure 2:**
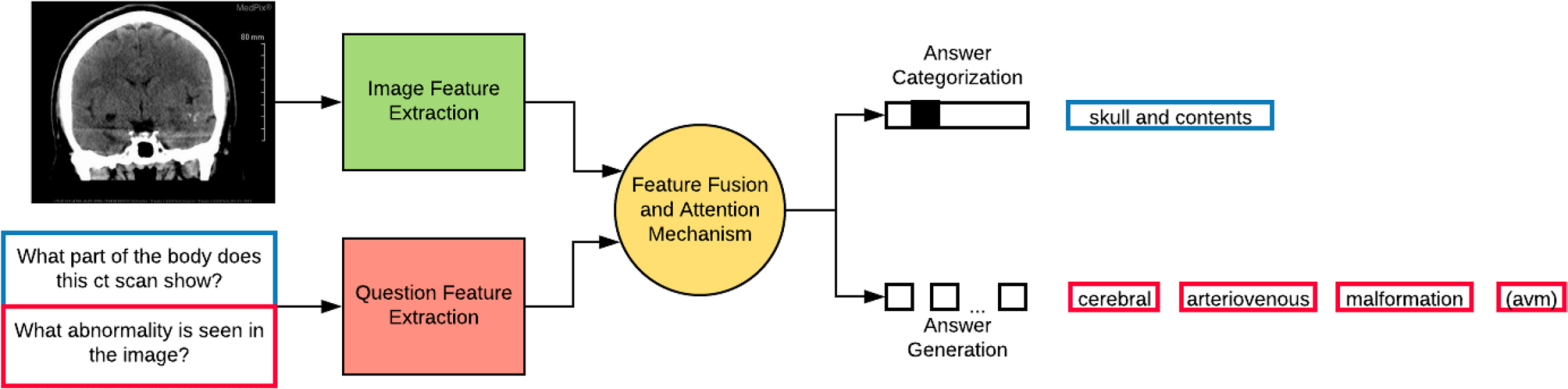
A high-level model design for the task of VQA. The model has four major components—image feature extraction, question feature extraction, feature fusion amalgamated with the attention mechanism, followed by answer categorization or generation depending on the task.

To address the above challenges, we propose *MedFuseNet*, an attention based multimodal deep learning model which learns representations by optimal fusion of the multimodal inputs using attention mechanism. Our *MedFuseNet* has four major components—*image feature extraction, question feature extraction, a feature fusion module,* and an *answer prediction module*. In addition, we employ attention modules to focus on the most relevant part of the medical images and questions. The answer prediction module has two submodules for answer categorization and answer generation tasks. For answer categorization task, *MedFuseNet* selects an answer from the set of possible answers while for answer generation task our model produces a meaningful sequence of words that answers the input question by utilizing a a full-fledged generative decoder. We conducted experiments on the MED-VQA 2019 dataset and PathVQA datasets, and show superior performance when compared to multiple VQA approaches including state-of-the-art attention-based VQA models. A few sample question-answer pairs from these datasets are shown in Fig. 1. The high-level illustration of our model is shown in Fig. 2.

 The major contributions of this paper are as follows:

- We propose *MedFuseNet*, an attention based multimodal deep learning model for answer categorization and answer generation tasks in medical domain VQA. We show that a LSTM-based generative decoder along with heuristics can improve our model performance for the answer generation task.
- We demonstrate state-of-the-art results on two real-world medical VQA datasets. In addition, we conducted an exhaustive ablation study to investigate the importance of each component in our proposed model.
- We study the interpretability of our *MedFuseNet* by visualizing various attention mechanisms used in the model. This provides a deeper insight into understanding the VQA capability of our model.

The rest of the paper is organized as follows. The “Related works” section  explores the existing methods for learning features from the multi-modal inputs, their fusion, and the existing models for VQA pertaining to real-world and medical VQA. The “Our proposed *MedFuseNet* model” section  presents the entire *MedFuseNet* framework, and the approach to tackling the VQA problem for the medical domain. This is followed by the comprehensive discussions of the experiments and the results in the “Experiments” section. The “Conclusion” section presents the conclusions and the future work.

## Related works

In this section, we first provide an overview of related works for VQA tasks for real-world and medical domains, and then discuss the related works on components of VQA approaches.

### Visual question answering

VQA for real-world domains has been a well-explored problem using various datasets such as DAQUAR^12^, VQA^13^, VQA 2.0^14^, and CLEVR^15^. There are mainly two lines of works in VQA: approaches that use attention mechanism, and approaches that do not use attention mechanism. Early works such as^16,17^ used simple concatenation of image-based and question-based features to obtain a representation of these multmodal inputs. These works obtained good results on VQA for natural images without using attention mechanism. Recent works such as^11,18–21^ used attention mechanism or attention modules to focus on the important parts of the image relevant to the question model, thus finding the correct and accurate answers. All these works were designed for VQA in natural images and trained on large datasets.

Researchers started exploring VQA in the medical domain recently with small medical VQA datasets such as RAD-VQA^22^, Indian Diabetic Retinopathy Image Dataset (IDRiD)^23^; and the ImageCLEF MED-VQA 2019 dataset^10^ released at ImageCLEF competitions has accelerated more research on this topic. The majority of works on VQA in the medical domain tried the VQA task as a classification problem^24–26^, i.e., build models for VQA answer categorization task. However, there have been limited research conducted on the answer generation task for medical VQA. Work in^27^ presented an approach to tackle both answer generation and answer categorization tasks. This work used a transformer model to generate a sequence of words for answer generation task. The authors of^28^ presented a different perspective on solving VQA for the medical domain by presenting a model that is more aware of the input question. However, all these works do not present a robust way to handle multimodal inputs for medical VQA tasks, and do not perform comparison of popular and state-of-the-art VQA models. Moreover, these works do not provide an interpretation of the VQA results which is important in medical domain. In our work, we address the limitations of the previous works by proposing a novel *MedFuseNet* and conduct experiments on two medical VQA datasets—MED-VQA^10^ (a radiology based VQA dataset) and PathVQA^29^ (a pathology based VQA dataset).

### VQA components

A typical VQA model contains image feature extraction, question feature extraction and a feature fusion component. We will now briefly discuss the related works for each of these components/modules.

#### Image representation learning

The superior performance of the Convolutional Neural Networks (CNN) in computer vision tasks has established CNN models as a reliable tool for robust feature representation from images. Generally speaking, the intermediate layer just before the output layer is used as the feature vector and popular models like VGGNet^30^, AlexNet^31^, DenseNet^32^, ResNet^33^ trained on large-scale image datasets such as ImageNet^34^ are used for image representation learning. That is, the features obtained from the intermediate layers of these pre-trained deep learning models provide a rich feature representation of the input image.

#### Textual representation learning

For textual data, there have been various strategies to represent the features. Word2Vec^35^, GloVe^36^, FastText^37^ are some of the word embedding algorithms that have been successful in obtaining a robust representation of the text at a word level. Sequential networks such as Recurrent Neural Networks (RNNs)^38^, Long-Short Term Memory (LSTM) networks^39^ have been then used to learn richer representations from these embeddings. BERT^40^ and XLNet^41^ have become the state-of-the-art models for many NLP tasks, and, hence, have been used for question feature extraction in VQA tasks.

#### Feature fusion techniques

The most intuitive way of combining the feature vectors is through the element-wise multiplication of vectors. However, due to the limited interaction of the elements of the two participating vectors, the outer product or the bilinear product of the two vectors is a better strategy to capture a robust and complete interaction of all the elements. Various fusion techniques relevant to VQA have been devised over time to maximize vector interactions and to reduce computational cost. These include Multimodal Compact Bilinear Pooling (MCB)^20^, Multimodal Low-rank Bilinear Pooling (MLB)^42^, Multimodal Tucker Fusion (MUTAN)^43^, Multimodal Factorized Bilinear Pooling (MFB)^44^. All these approaches are build on similar idea of making the bilinear pooling of two vectors computationally feasible.

Our work leverages the recent advances of the above components, and we propose a novel multimodal attention model (described in detail in the next section) for medical VQA tasks.

## Our proposed *MedFuseNet* model

In this section, we will first define the problem statements for VQA answer categorization and answer generation tasks for the medical domain, and then discuss our proposed *MedFuseNet* model and it’s components in detail.

### Problem definitions

Using the notations mentioned in Table 1, we define the medical VQA answer categorization and generation tasks as follows:

#### Definition 1

**Answer Categorization task.** Given a medical image *v*, an associated natural language question *q*, the aim of this task is to produce the answer $\tilde{a}$ from a possible set of answers ${\mathscr {A}}$, where the ground truth answer is represented by *a*. This can be formulated as follows

1 $$\begin{aligned} \tilde{a} = \mathop {{\mathrm{argmax}}}\limits \limits _{a \in {\mathscr {A}}} P(a|v,q;\Theta ) \end{aligned}$$

where $\Theta$ is the set of model parameters, *v* is the input radiology scan, and *q* is the natural language question associated with the image in Eq. (1).

#### Definition 2

**Answer Generation task.** Given a medical image *v*, a natural language question associated with the image *q*, the aim of this task is to generate a sequence of words $\tilde{a} = [\tilde{a}_1, \dotsc , \tilde{a}_i]$, where the ground truth answer is represented by $a = [a_1, \dotsc , a_j]$, where $\tilde{a}_1, \dotsc , \tilde{a}_i$ and $a_1, \dotsc , a_j$ belong to the answer word vocabulary $W_{{\mathscr {A}}}$. This can be represented as

2 $$\begin{aligned} {[}\tilde{a}_1, \dotsc , \tilde{a}_i] = \mathop {{\mathrm{argmax}}}\limits \limits _{a_1, \dotsc , a_j \in W_{{\mathscr {A}}}} P(a_1, \dotsc , a_j|v,q;\Theta ) \end{aligned}$$

where $\Theta$ is the set of model parameters, *v* is the input radiology scan, and *q* is the natural language question associated with the image. We define the VQA answer generation task as generating a sequence of words from the answer word vocabulary $W_{{\mathscr {A}}}$ as shown in Eq. (2).

For the answer categorization task, we use a softmax cross-entropy loss function to find the error in the answer prediction of the model, and this loss is given by:

3 $$\begin{aligned} {\mathscr {L}}(a, \tilde{a}) = \sum _{i} - p(a_i) \log (p(\tilde{a}_i)) \end{aligned}$$

where $p(\tilde{a}_i)$ is the probability of $\tilde{a}_i$ being the answer, and $p(a_i)$ is the probability of $a_i$ being the ground-truth answer. For the answer generation task, we use the cross-entropy loss defined in Eq. (3) to calculate the error in predicting each word of the generated answer from the word vocabulary $W_{{\mathscr {A}}}$.

### Overview of the *MedFuseNet* model

Our *MedFuseNet* is an attention based multimodal deep learning model which learns representations by optimal fusion of the inputs using attention mechanism. *MedFuseNet* consists of four main components—Image feature extraction, question feature extraction, feature fusion, and answer prediction. The image feature extraction component takes medical image *v* as input and will output an image feature vector ${\hat{v}}$. Similarly, the question feature extraction component will generate the feature vector ${\hat{q}}$ for the input question *q*. The feature vectors are then combined to form *z*. The combined vector *z* and attention modules are used to predict the answer depending on the VQA task—answer categorization or answer generation.

**Table 1:** Notations used in this paper.

Notation	Description
*v*	Input image
${\hat{v}}$	Image feature vector
${\hat{v}}_e$	Attended image feature vector
*q*	Input question
${\hat{q}}$	Question feature vector
${\hat{q}}_e$	Attended question feature vector
*z*	Combined feature vector
$d_i$	Attention output for the $i^{th}$ step of the decoder
$h_i$	LSTM output for the $i^{th}$ step of the decoder
*g*	Number of attention glimpses
*a*	Actual answer
$\tilde{a}$	Predicted answer
$[a_1, \dotsc , a_j]$	Actual answer sequence
$[\tilde{a}_1, \dotsc , \tilde{a}_i]$	Predicted answer sequence
$\Theta$	Model parameters
${\mathscr {L}}$	Loss function
${\mathscr {A}}$	Possible set of answers
$W_{{\mathscr {A}}}$	Vocabulary of words in answers
$\circ$	Inner product operation
$N_b$	Batch size
${\mathscr {E}}_v$	Image Attention
${\mathscr {E}}_q$	Question Attention
${\mathscr {E}}_d$	Decoder Attention

### Components of *MedFuseNet* model

Here, we will describe in detail the different components of our *MedFuseNet*.

#### Image feature extraction

The feature learning from images has been an active research area for decades. An intermediate layer of a CNN captures the features of the image at varying levels of abstraction. While the shallow layers represent a more elementary level of features, the deeper layers encapsulate a more abstract set of features. Exploiting this interpretation, generally, the penultimate layer just before the output layer of CNN is used to extract a feature vector for an input image. As described in “Image representation learning” section, VGGNet-16^30^, DenseNet-121^32^ , and ResNet-152 models^33^ can be used for image feature extraction. Since the medical images are complex compared to the standard real-world images, models like DenseNet-121 and ResNet-152 which have skip connections, provide more robust feature representations through deeper convolutional layers. Due to the superior performance of ResNet-152 over the other two, our *MedFuseNet* model uses it as the image feature extraction module to learn representations of medical images. In our experiments and ablation studies described in the “Experiments” section, we have used all these models—VGGNet-16, DenseNet-121, and ResNet-152 models for learning medical image feature representations. It should be noted that the intermediate output from the last convolutional block of each of these model was used as the feature representation of the medical image, and these models were pre-trained on the ImageNet dataset.

#### Question feature extraction

As discussed earlier in “Textual representation learning” section, word embeddings form the primary method for expressing the underlying context of natural language. However, they are insufficient and do not capture the context properly. While modeling the feature representation of the natural text, it is necessary that we appropriately capture the positional semantics of each word and not just the word-level semantics. The state-of-the-art NLP models such as BERT and XLNet can capture positional and word-level semantics and are thus better at representing the features of the input question. The primary idea behind these models is to learn an exhaustive textual representation of the question. Our *MedFuseNet* model uses BERT for the question feature extraction. Also, note that in our experiments and ablation studies described in the “Experiments” section, we have used both BERT and XLNet for question feature extraction, and we noticed that BERT generally obtains better results than XLNet. The pre-trained versions of both these models were used for the question feature extraction of the question.

#### Feature fusion techniques

An intuitive way to combine multiple feature vectors is by concatenation. However, such a simple concatenation does not capture the feature interactions. Another common way of combining the multiple feature vectors is through the inner product or the element-wise multiplication of the vectors. However, due to the limited interaction of the elements of the two vectors in the inner product, it is considered a primitive strategy for feature fusion. The outer product or the bilinear product of the two vectors is a better strategy as it can capture a robust and complete set of interactions of all the feature vector elements. A simple bilinear model for two vectors $v \in {\mathbb {R}}^{m}$ and $q \in {\mathbb {R}}^{n}$ is shown in Eq. (4).

4 $$\begin{aligned} z_{i} = v^{T}W_{i}q \end{aligned}$$

where $W_{i} \in {\mathbb {R}}^{m \times n}$ and $z_{i} \in {\mathbb {R}}^{o}$. Thus, the model needs to learn the parameter matrix $W = [W_{1}, \dotsc , W_{o}] \in {\mathbb {R}}^{m \times n \times o}$ which is typically computationally expensive for large values of *m*, *n*,  and *o*. For example, if $m=1024, n=1024, o = 512$, then the number of parameters in the projection matrix *W* will be $\sim 530$ million parameters, and computationally expensive and infeasible to learn it. Recently, various works such as Multimodal Compact Bilinear (MCB) Pooling^20^, Multimodal Tucker Decomposition (MUTAN)^45^, and Multimodal Factorized Bilinear Pooling (MFB)^44^ have been proposed to address this problem. Each of these techniques simplify the process of Bilinear Pooling by presenting a way to decompose the outer product projection matrix *W*. Due to the simplicity of the MFB algorithm, ease of implementation and high convergence rate, our *MedFuseNet* uses it over other approaches for multimodal feature fusion. In addition, to avoid our *MedFuseNet* model from converging to a local minima, the output of the MFB module is normalized using power normalization and L-2 normalization^44^. Our experiments and ablation studies described in the “Experiments” section also support that MFB fusion strategy typically performs better than MCB and MUTAN fusion strategies.

#### Attention mechanisms

A typical model for VQA first extracts the feature vectors from multiple modalities (image and question text), and then combines the vectors using any one of the above-stated fusion techniques, and then predicts the answer from the fused vector. However, questions that are very specific to the input image require a more specific context of the image. This is where attention mechanisms prove to be useful as they help to focus on the most relevant parts of the input. Our model, *MedFuseNet*, uses two types of attention mechanisms namely *Image Attention* and *Image-Question Co-Attention*—to capture the context in medical images that are relevant to answer the question. Below, we describe these attention mechanisms and the role played by them in the training of our *MedFuseNet*.

**Figure 3:**
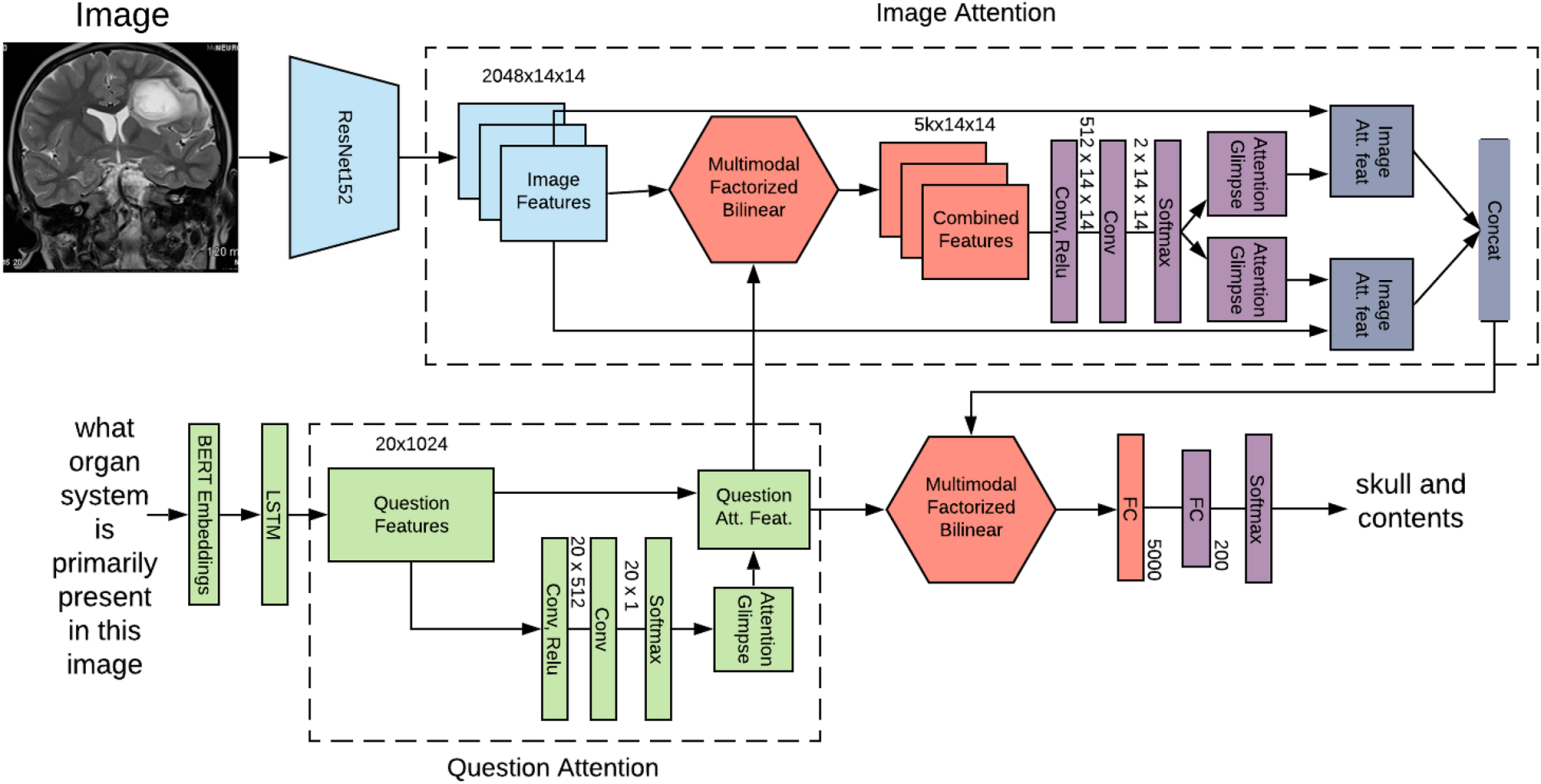
Our end-to-end framework for Medical Visual Question Answering for answer categorization. It takes the medical image and the associated question as the inputs, followed by the feature extraction. The question features are further processed using the question attention mechanism. The attended question features and the image features are then passed through the image attention mechanism to get the attended image features. These attended vectors are finally combined using MFB to build the answer classification module.

**Image Attention:** The image attention mechanism aims at spanning the attention of the *MedFuseNet* model to the most relevant part of the image on the basis of the input question. This establishes a correlation between the multimodal input and helps the model converge faster. The image attention mechanism combines the feature fusion technique with the attention maps to come up with the attended image feature vector as given in lines 20-30 of Algorithm 1. Firstly, the image features ${\hat{v}}$ and question features ${\hat{q}}$ are combined using the fusion technique (line 21). The attention maps are then computed from this combined feature vector (lines 22-23). The input image features ${\hat{v}}$ are then overlaid with the attention glimpses (lines 24-28) to get the attended image feature vector ${\hat{v}}_e$. The pictorial representation of the algorithm is shown in Fig. 3.

**Image-Question Co-Attention:** The image attention mechanism focuses on the significant parts of the image, however, it takes the entire question into consideration. A co-attention mechanism exploits the intuition that the key parts of the question can be solely computed for the question which can further be used to enhance the image attention. So, our *MedFuseNet* model first computes the attended question feature vector ${\hat{q}}_e$ using the Question attention mechanism ${\mathscr {E}}_q$ as shown in Fig. 3. It then uses this attended vector as an input to the image attention mechanism as described in Algorithm 1 from lines 8-18, instead of question feature vector ${\hat{q}}$.

### *MedFuseNet* model for medical VQA tasks

As described in the various components of our *MedFuseNet* model, our approach aims at maximizing the performance for answer prediction and minimizing the model complexity. The three main components of our model include (a) pre-trained ResNet-152 for image feature extraction, (b) pre-trained BERT for question feature extraction, and (c) MFB for feature fusion. Moreover, *MedFuseNet* uses attention techniques so that the model focuses only on the most relevant parts of the image and the question while predicting the answer. The pictorial representation of the model is shown in Fig. 3. 
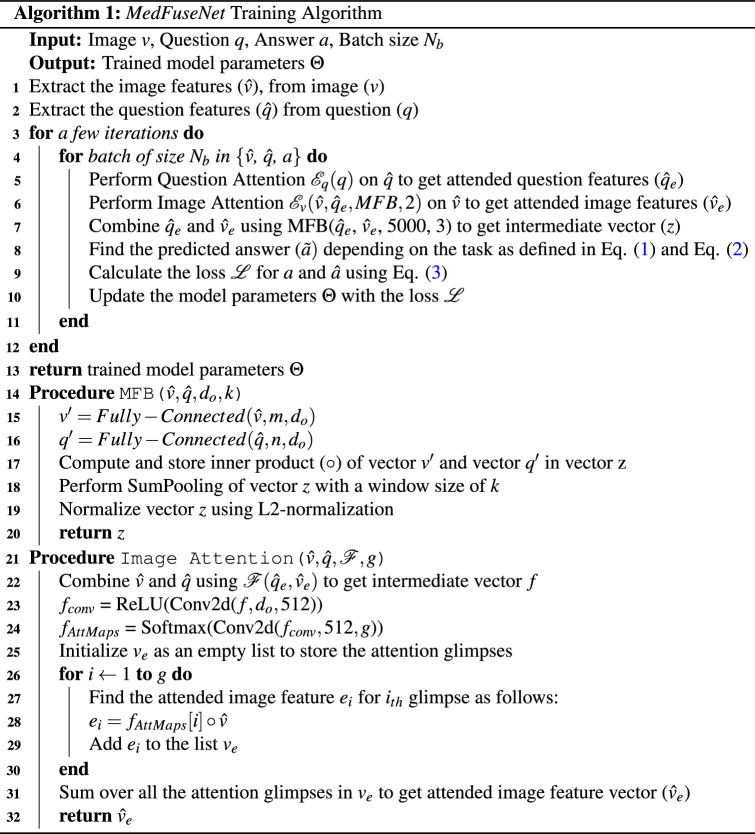


Our *MedFuseNet* model tackles all the challenges specific to the VQA in medical domain as stated in the “Introduction” section. The following aspects help in boosting the performance of *MedFuseNet* for medical VQA:

- ResNet and BERT models are pretrained on very large datasets, and they provide a much better generalization for the features by the virtue of transfer learning.
- Due to the simplistic implementation of MFB, it reduces the complexity of calculating the outer product to a large extent, while conserving the information from the fusion of the two modalities. This reduces the computation of model parameters and works well for the limited MED-VQA datasets.
- The attention and co-attention mechanisms help in reducing the attention span of the model to the significant parts of the input, thus, reducing the search space for the model.

#### Answer categorization

As shown in Algorithm 1 (lines 1–12), the *MedFuseNet* first extracts the feature vectors ${\hat{v}}$ and ${\hat{q}}$ for input image *v* and question *q*, respectively. This is followed by the computation of the attended question features ${\hat{q}}_e$ using question attention mechanism ${\mathscr {E}}_q (q)$. Then, it uses the Image Attention mechanism ${\mathscr {E}}_v$ as explained in Algorithm 1 (lines 20-30) to get the attended image features ${\hat{v}}_e$. ${\hat{v}}_e$, and ${\hat{q}}_e$ are then combined using MFB (lines 13-19) to get vector *z*. For answer categorization VQA task, a classification model is then built over *z* to find the loss and update the model parameters $\Theta$.

#### Answer generation

**Figure 4:**
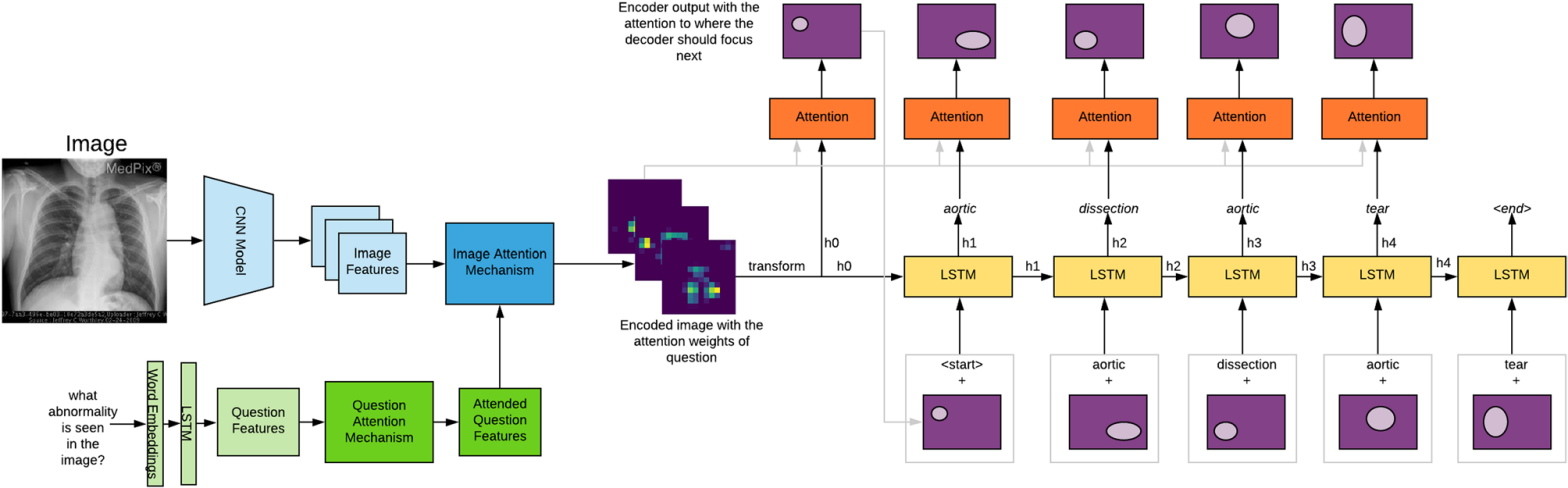
The architecture used for the answer generation task. This module takes the image and the question as the input. It generates the feature vectors for both and produces the combined vector after fusing them using MFB as part of the image-question co-attention mechanism. This is followed by an LSTM-based decoder to generate the answer. The two major components of this decoder are—the attention mechanism and teacher forcing. The attention mechanism helps the model in focusing on various parts of the image while generating a word, and the teacher enforcing helps the model converge faster.

As described in definition 2, the problem of answer generation is not a straightforward task as we need to generate a meaningful sequence of words from the answer word vocabulary $W_{{\mathscr {A}}}$ to predict the answer. Hence, we propose and develop a more sophisticated model for the answer prediction task. Our answer prediction module shown in Fig. 4 consists of a LSTM-based decoder model which uses the fused feature vector for answer prediction. Our decoder model is inspired by the work presented in^46^. The novel characteristics of our answer generation decoder module are as follows:

- **Teacher Forcing:** Due to the inherent complexity of the task of sequence generation, the decoder is susceptible to a slower convergence rate. Moreover, the limited amount of data in the medical domain may cause more hindrance to the model convergence rate. Thus, to increase the learning rate of the model, we use Teacher Forcing^47^. As shown in Fig. 4, we pass to the decoder the ground-truth word for the $i^{th}$ time-step to predict the next word at $(i+1)^{th}$ time-step.
- **Attention Mechanism:** To make each LSTM step prediction more accurate, we also incorporate the attention mechanism in the decoder. We use the output of the ${i-1}^{th}$ time-step to span the focus of the model on those parts of the image feature vector ${\hat{v}}_e$ that have already been answered. This helps the model to guide its search for the $i^{th}$ word in the generated answer more precisely.
- **Beam Search:** During inference, we use Beam Search heuristic^48^ to avoid the model from greedily generating the answer by choosing the best word at each decoding step.

Before generating the answer sequence using the decoder, we fuse the input image *v* and question *q* to get the attended image features ${\hat{v}}_e$ as described in the Image Attention procedure of the Algorithm 1. This obtained vector ${\hat{v}}_e$ is passed to the decoder to generate the answer. As shown in Algorithm 2, ${\hat{v}}_e$ is first used to initialize the states of the LSTM (line 1). Following this, for the *i*th step of the decoder, we concatenate the output $d_{i-1}$ of the attention mechanism ${\mathscr {E}}_d$ for $(i-1)$th step with the *i*th word in the ground truth answer, that is $a_i$, as shown in line 3 in Algorithm 2. This concatenated vector is then fed to the LSTM cell to get $h_i$ which is also $\tilde{a}_i$, the *i*th word in the predicted answer (lines 4–5 in Algorithm 2). The vectors $h_i$ and ${\hat{v}}_e$ are then fed to the attention mechanism (lines 6–7 in Algorithm 2). The pictorial representation of the end-to-end model for answer generation is shown in Fig. 4. 
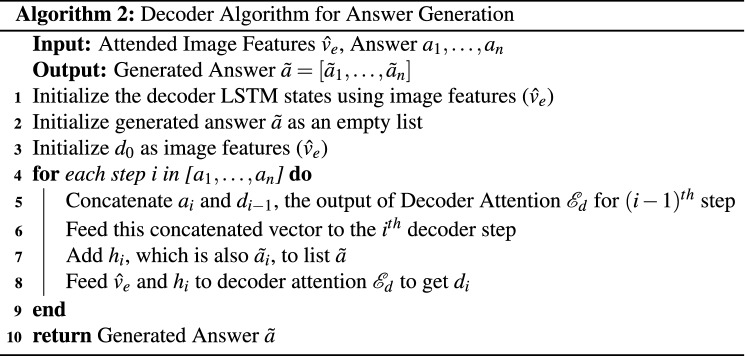


## Experiments

We conducted several experiments on two real-world medical VQA datasets to compare the performance of our proposed model with the state-of-the-art and many popular VQA approaches. Our experiments will answer the following key questions:

- How does *MedFuseNet*, our proposed model, perform w.r.t. the state-of-the-art VQA models for the answer categorization and answer generation tasks?
- Can we visualize and explain the results of our proposed model?
- What is the impact of different attention mechanisms on model performance?
- How good are the answers generated by the proposed model in terms of BLEU scores?

To answer the above questions, we first describe the datasets used for the answer categorization and answer generation tasks, and then describe in detail the dataset processing, implementation, evaluation metrics, and baseline models for comparison.

### Datasets for answer categorization task

#### MED-VQA

This dataset was released at the ImageCLEF 2019 MED-VQA challenge^10^, and it contains 4200 medical images and medical questions associated with each image. Examples are shown in Fig. 1 and the data distribution is shown in Table 2. Each question belongs to one of the three categories—Modality, Plane, and Organ. In total, there are 3825 image-question-answer triplets for each category. The three question categories are as follows:

- **Modality:** This category pertains to the modality of the input medical image, and the question-answer pairs belong to 36 classes.
- **Plane:** This category pertains to the plane in which the medical image was taken/scanned, and the question-answer pairs with planes come from 16 classes/categories.
- **Organ System:** This category describes the organ system captured in the medical image, and the question-answer pairs belong to 10 unique organ systems.

The maximum question length for the three question categories combined is 13 words and the average question length is around 8 words. The combined vocabulary of the questions contains about 100 words.

**Table 2:** Train, validation, and test splits for the yes-no type question-answer pairs in MED-VQA dataset.

Split	Modality	Plane	Organ
Train	3200	3200	3200
Validation	500	500	500
Test	125	125	125

#### PathVQA

This is the VQA dataset^29^ on pathology images prepared using a novel pipeline from the captions of the images in the medical textbooks. The dataset has 9000+ medical images and 47,000+ question-answer (QA) pairs. We use only the ‘yes/no’ type question-answer pairs for the answer categorization experiments in this paper. The dataset is divided into train, validation, and test splits. All the three splits have a fairly well distributed yes-no types question answers with almost a 1:1 proportion. The details of the dataset is presented in Table 3.

**Table 3:** Train, validation, and test splits for the yes-no type question-answer pairs in PathVQA dataset.

Split	Medical Images	‘Yes’ type QA Pairs	‘No’ type QA Pairs
Train	4271	9305	9163
Validation	1176	2359	2335
Test	942	1874	1853

### Datasets for answer generation task

#### MED-VQA

Other than the three categories mentioned in the “MED-VQA” section, there is one additional class of question in the ImageCLEF 2019 MED-VQA challenge^10^ dataset—‘abnormality’. The answers for this question category are open-ended, and they describe the abnormality present in the medical image/scan. Answering these types of questions is typically more useful to the healthcare providers as it can help them in getting a second opinion on some critical cases. We consider the question-answer pairs for the abnormality question category as the dataset for our answer generation task for the MED-VQA dataset. In total, we have 3817 question-answer pairs for abnormality question category. The combined word vocabulary of the answers is 2109 words, out of which 756 words have an occurrence of one in the entire dataset. This poses a greater challenge to the model the answer generation for this skewed dataset. The average length of an answer is 2.63 words and the average length of a question is $\sim 7$ words.

#### PathVQA

As discussed in the “PathVQA” section, PathVQA is a dataset about the question-answers related to pathology images. Apart from the yes-no type question-answers, it also has a great proportion of open-ended answer type questions. For the set of experiments related to the answer generation task, we subsample a dataset from open-ended answer type questions of the PathVQA dataset. To assure that the data is not skewed, we sample only those answers which have a frequency of at least 5 in the entire dataset. This gives us a total of 6770 question-answer pairs with 4192 unique cases. The vocabulary size of the answers is about 480 words. The average number of words in an answer is 2.76 words. The average question length is $\sim 6$ words.

#### Dataset preprocessing

For all the datasets described above, the medical images were resized to be of the same dimension of $224 \times 224 \times 3$. This was done as most of the well-accepted pre-trained models take the input in this dimension. For each question, we first tokenized using the NLTK library in python^49^. Then, the question vocabulary was prepared and the tokens in the vocabulary were enumerated, which was used to convert the question to a list of numbers. The questions were also padded to make them all of the same lengths.

### VQA baseline models for comparison

We establish the superior performance of *MedFuseNet* by comparing it with the five baselines for the answer categorization task. Three of the baselines are attention-based VQA models, while the other two are popular VQA models.

- **VIS + LSTM**^50,51^—This is a relatively simpler model that uses vanilla LSTM for question feature extraction, and a CNN model for image feature extraction. The LSTM of the question feature was initialized using the image features. The last output of LSTM was used to predict the answer by using a dense-layer.
- **Deeper LSTM + Norm. CNN (d-LSTM + n-I)**^52^—This model again uses a VGG16 for image feature extraction and a 2-layer LSTM model for question features. The two feature vectors are then combined using a simple element-wise multiplication to get the output vector.
- **Stacked Attention Networks (SAN)**^18^—SAN is an attention-based VQA model, and it uses multiple attention layers to refine the search space of the two feature vectors. It uses VGG16 based image features and CNN to extract the features of the question text. It then stacks attention layers over image vector and then applies an array of attention vectors on the question to obtain the final combined feature vector.
- **Hierarchical Co-attention (HiCAt)**^19^—This is another attention-based VQA model. The image features are CNN-based while the question features are obtained by performing 1-D convolution over a word-embedding to get a hierarchy of the text. Two attention schemes are used in this work: parallel attention and alternating co-attention. In parallel attention, the model captures the attention of both vectors simultaneously while in the latter one, attention is alternated between the feature vectors of the two inputs.
- **Bilinear Attention Networks (BAN)**^21^—BAN is a state-of-the-art VQA method that combines the attention mechanism with the feature fusion technique to maximize the model performance. It uses a modified version of MFB model for feature fusion wherein the attention mechanisms come into action during feature combination. It uses FasterRCNN features with the aim of using localized feature fusion instead of using a global feature vector.

For the task of answer generation, there are no suitable baselines that are appropriate for comparison. Hence, we use BAN as one of the baseline comparison models and plug-in a decoder into the model architecture to make it compatible for the answer generation task. This decoder is a simple LSTM-based model. We also incorporate teacher forcing method in this decoder to help the model converge faster.

### Evaluation metrics

For evaluating the performance of the model in all the datasets discussed in “Datasets for answer categorization task” and “Datasets for answer generation task” sections, we use stratified 5-fold cross-validation after combining the training, the validation, and the testing splits. This helps in understanding the generalization capability of the proposed model.

#### Answer categorization task

We use three metrics to evaluate the performance of the model—*Accuracy*, *Area Under Curve—Receiver Operator Characteristics (AUC-ROC)*, and *Area Under Curve—Precision-Recall Curve (AUC-PRC)*^53^ for the task of answer categorization. Accuracy is the primary metric used for any classification/categorization task and it quantifies the performance of the model in distinguishing between various classes. However, accuracy scores can be misleading for the data with imbalanced classes, as in the case of the MED-VQA dataset. So, we also calculate the AUC-ROC and AUC-PRC. AUC-ROC is defined by the area under the Receiver Operating Characteristics (ROC) Curve. A ROC curve describes the ability of the model to separate between various classes by plotting False Positive Rate (FPR) on X-axis and True Positive Rate (TPR) on the y-axis. Higher the area under the curve the better the performance of the model will be. Similarly, AUC-PRC is the area under the curve with Precision on Y-axis and Recall on X-axis. Higher the value AUC-PRC the better the performance. These metrics help us gauge the performance of the model with respect to the answer prediction task considering the class imbalance as well. For the PathVQA dataset, we use only the accuracy as a metric to evaluate the performance of the models as the classes are fairly balanced with an equal proportion of yes and no type answers.

#### Answer generation task

To evaluate the answer generation capability of our model, we use generated sequence evaluation metrics such as Bilingual Evaluation Understudy (BLEU) score^54^. BLEU score calculates the similarity of the reference (ground truth answer) and the hypothesis (predicted answer) at an *n*-gram level. Thus, it is a very useful metric for comparing two sequences or sentences. Specifically, we use BLEU-1, BLEU-2, and BLEU-3 scores to compare the sequences at 1-gram, 2-gram, and 3-gram levels, respectively. Apart from the BLEU score, we also compute the F-1 score of the generated answer. In terms of sequence generation, the F-1 score gives a good indication about the performance of the model in generating the correct words. We use the NLTK library in Python for calculating these metric scores.

### Implementation details

We have implemented all the components of *MedFuseNet* using PyTorch^55^. The image feature extraction was developed using pre-trained models available in Keras^56^. Embedding-as-a-Service^57^ was used for extracting the features for question from the pre-trained BERT and XLNet models. The size of each question was made uniform with 20 tokens. The size of the combined feature vector is set to be 16, 000 for MCB, 5000 for MFB and MUTAN. These feature sizes were chosen as suggested by the authors of the respective works. The number of LSTM steps were fixed as 1024. For attention modules, 2 attention glimpses were used. We used the ADAM optimizer^58^ with $\beta _1 = 0.9$ and $\beta _2 = 0.999$ with a learning rate of 0.001. Cross-Entropy loss was used to calculate the error between the predicted and the actual answer. The model was trained for 100 epochs with a batch-size of 32. We used the Scikit-Learn package^59^ to calculate the performance metrics. The codes for implementing fusion techniques were obtained from MCB^60^, VQA PyTorch^61^, OpenVQA^62^ github repos.

The implementation of the decoder part of our *MedFuseNet* is done in PyTorch. The code for the same is adapted from Image-Captioning-Pytorch^63^. We used the ADAM optimizer with a learning rate of $10e^{-4}$ and Cross-Entropy loss function to calculate the sequence generation loss. The model was trained for 30 epochs with a batch-size of 32. The BLEU-scores were evaluated using the NLTK Module^64^ .

For the first three baselines, the code was adapted from SAN-VQA^65^. For HiCAt, the code was adapted from HiCAt^66^. The code for BAN was adapted from ban-vqa^67^. The FasterRCNN features for BAN were extracted using the code available in FasterRCNN-Visual Genome^68^. In order to ensure reproducibility of our work, we have publicly released the source code of the proposed MedFuseNet model in PyTorch at this URL: https://github.com/dhruvsharma15/MEDVQA.

### Experimental results

#### Comparisons for answer categorization task

We quantitatively evaluate the performance of *MedFuseNet* and compare it with the baseline models described in the “VQA baseline models for comparison” section for the tasks of answer categorization and answer generation.

The performance values of each model for answer categorization task with the MED-VQA dataset are summarized in Table 4. Comparing the accuracy scores for all three question categories, we can clearly see that *MedFuseNet* outperforms the BAN model. *MedFuseNet* achieves accuracy scores of **0.840** for category 1 (Modality), **0.780** for category 2 (Plane), and **0.746** for category 3 (Organ). Whereas the BAN model is more competitive to *MedFuseNet* model for category 3, while the BAN model under-performs our model for category 1 by 2 percent and category 2 by 1.4 percent. In terms of AUC-ROC, BAN model outperforms *MedFuseNet* with a scores of **0.961** for category 1, **0.929** for category 2, while *MedFuseNet* leads with a score of **0.800** for category 3. For AUC-PRC scores, *MedFuseNet* outperforms all the baselines. This superior performance of *MedFuseNet* demonstrates that baseline VQA models (like VIS + LSTM and Deeper LSTM + normalized CNN) may be insufficient to capture the underlying patterns in image question pairs. On the other hand, the attention mechanisms present in SAN and Hierarchical Co-Attention model might make the architecture more complex which requires more data to learn the parameters, and then leads to poor AUC-PRC scores. The AUC-PRC scores in Table 4 clearly indicate that simpler models like VIS + LSTM outperform the attention-based models. Although, BAN proves to be a strong contender, *MedFuseNet* quantitatively outperforms all the baselines and BAN model, as it is designed to handle limited amount of data in the medical domain. Another observation worth noting is the difference in the AUC-ROC and AUC-PRC scores of our *MedFuseNet* as shown in Table 4. This indicates that our *MedFuseNet* is comparably better in detecting true negatives, due to comparably high AUC-ROC score, than detecting true positives, because of the low AUC-PRC score, which can be attributed to the high class-imbalance.

**Table 4:** Comparison of *MedFuseNet* with the baseline models on MED-VQA answer classification dataset.

Methods	Accuracy			AUC-ROC			AUC-PRC		
	Modality	Plane	Organ	Modality	Plane	Organ	Modality	Plane	Organ
VIS + LSTM^50^	0.704(0.012)	0.701(0.017)	0.652(0.020)	0.899(0.012)	0.851(0.011)	0.775(0.015)	0.478(0.024)	0.453(0.022)	0.456(0.025)
d-LSTM + n-CNN^52^	0.723(0.014)	0.719(0.018)	0.672(0.022)	0.909(0.010)	0.862(0.014)	0.777(0.017)	0.474(0.025)	0.459(0.023)	0.450(0.027)
SAN^18^	0.669(0.013)	0.729(0.015)	0.669(0.023)	0.926(0.011)	0.870(0.011)	0.783(0.015)	0.459(0.025)	0.415(0.023)	0.406(0.026)
HiCAt^19^	0.760(0.010)	0.740(0.015)	0.668(0.018)	0.929(0.011)	0.869(0.010)	0.797(0.014)	0.468(0.023)	0.431(0.025)	0.430(0.028)
BAN^21^	0.820(0.011)	0.766(0.016)	**0.750(0.014)**	**0.961(0.010)**	**0.929(0.009)**	0.800(0.016)	0.600(0.024)	0.521(0.022)	0.456(0.025)
*MedFuseNet*	**0.840(0.010)**	**0.780(0.017)**	0.746(0.015)	0.942(0.010)	0.901(0.010)	**0.800(0.013)**	**0.618(0.023)**	**0.526(0.024)**	**0.510(0.023)**

For the PathVQA dataset with yes-no type answers, the accuracy scores of the baselines and *MedFuseNet* are presented in Table 5. Since the PathVQA dataset is balanced for yes and no type answers, we only use the accuracy score as the metric to compare the performance of different VQA models. As shown on Table 5, our *MedFuseNet* outperforms all the other VQA approaches and obtains an accuracy score of **0.636**. Amongst other baseline methods, we can observe that the performance of SAN^18^ and Hierarchical Co-Attention Networks^19^ is competitive, while that of BAN^21^ is relatively lower. This could be attributed to the fact that the answer categorization task for PathVQA might not be inherently complex to justify the need for more complex models. Moreover, the performance of the BAN is highly dependant on the bounding boxes extracted from the pre-trained FasterRCNN model. These bounding boxes might not always be informative since the FasterRCNN model is pre-trained using real-world images dataset like Visual Genome^69^ (and not fined-tuned for medical images). Thus, using BAN for pathological image categorization might provide misleading results.

**Table 5:** Comparison of *MedFuseNet* with the baseline models on PathVQA yes-no answer type dataset.

Methods	Accuracy
VIS + LSTM^50^	0.603(0.025)
d-LSTM + n-CNN^52^	0.607(0.021)
SAN^18^	0.627(0.023)
HiCAt^19^	0.629(0.018)
BAN^21^	0.604(0.021)
*MedFuseNet*	**0.636(0.020)**

#### Comparisons for answer generation task

The performance comparisons for the answer generation task on the MED-VQA abnormality category data and the open-ended answer type questions in PathVQA dataset is summarized in Table 6. For the MED-VQA dataset, we observe that *MedFuseNet* with the decoder performs better than the BAN model (with Decoder) for the metrics of BLEU-1 and BLEU-3 scores, while BAN (with Decoder) has better performance in terms of BLEU-2 and F-1 scores. This shows that two models compare favorably on this dataset. As there are 2–3 words on an average in the answer of the MED-VQA dataset, we do not have a clear winner since *MedFuseNet* is marginally better at a 3-gram level while BAN (with Decoder) performs better at answer generation evaluation at the 2-gram level. For the open-ended question-answer pairs of the PathVQA dataset, our *MedFuseNet* with the decoder significantly outperforms the state-of-the-art BAN model with decoder. Our *MedFuseNet* obtains a BLEU-1 score of **0.605**, BLEU-2 score of **0.303**, BLEU-3 score of **0.073**, and an F-1 score of **0.381** for on this dataset.

**Table 6:** Comparison of *MedFuseNet* with the baseline models on answer generation dataset.

Dataset	Method	BELU-1	BLEU-2	BLEU-3	F-1
MED-VQA	BAN + Decoder	0.266(0.015)	**0.083(0.008)**	0.013(0.002)	**0.274(0.012)**
	*MedFuseNet* + Decoder	**0.276(0.019)**	0.076(0.005)	**0.016(0.002)**	0.229(0.012)
PathVQA	BAN + Decoder	0.542(0.023)	0.216(0.023)	0.054(0.008)	0.378(0.009)
	*MedFuseNet* + Decoder	**0.605(0.021)**	**0.303(0.027)**	**0.073**(0.007)	**0.381(0.009)**

These experiments on the two real-world datasets show that our *MedFuseNet* with a decoder works well for the answer generation task. It should be noted that our contribution is the integration of decoder to our *MedFuseNet* model, and that this decoder is flexible and can be incorporated into any other VQA model such as BAN as shown in our comparison experiments.

**Table 7:** Performance metric scores for the ablation study experiments on MED-VQA dataset.

Question Category	Image Feature	MCB		MUTAN		MFB	
		BERT	XLNet	BERT	XLNet	BERT	XLNet
*Accuracy*							
Category 1 Modality	VGG16	0.718(0.019)	0.697(0.018)	0.751(0.016)	0.686(0.019)	0.805(0.012)	0.680(0.019)
	DenseNet121	0.704(0.015)	0.675(0.019)	0.768(0.014)	0.688(0.021)	0.813(0.014)	0.675(0.020)
	ResNet152	0.731(0.014)	0.663(0.017)	0.783(0.018)	0.716(0.017)	**0.840(0.011)**	0.701(0.018)
Category 2 Plane	VGG16	0.706(0.018)	0.697(0.016)	0.750(0.017)	0.605(0.022)	0.749(0.014)	0.629(0.019)
	DenseNet121	0.719(0.016)	0.643(0.018)	0.754(0.016)	0.643(0.017)	0.757(0.011)	0.655(0.021)
	ResNet152	0.712(0.015)	0.659(0.019)	0.763(0.015)	0.693(0.019)	**0.780(0.010)**	0.735(0.016)
Category 3 Organ System	VGG16	0.718(0.018)	0.625(0.015)	0.785(0.012)	0.683(0.016)	**0.798(0.011)**	0.692(0.019)
	DenseNet121	0.753(0.013)	0.630(0.018)	0.774(0.015)	0.696(0.018)	0.774(0.012)	0.720(0.016)
	ResNet152	0.669(0.016)	0.672(0.013)	0.705(0.016)	0.649(0.019)	0.746(0.010)	0.682(0.015)
*AUC-ROC*							
Category 1 Modality	VGG16	0.845(0.011)	0.697(0.016)	0.896(0.010)	0.710(0.015)	**0.954(0.011)**	0.738(0.015)
	DenseNet121	0.854(0.013)	0.675(0.018)	0.898(0.010)	0.659(0.014)	0.934(0.010)	0.703(0.016)
	ResNet152	0.861(0.012)	0.703(0.018)	0.906(0.011)	0.740(0.017)	0.942(0.013)	0.700(0.014)
Category 2 Plane	VGG16	0.833(0.012)	0.697(0.018)	0.866(0.011)	0.718(0.017)	0.899(0.013)	0.729(0.014)
	DenseNet121	0.832(0.013)	0.743(0.017)	0.867(0.012)	0.801(0.013)	0.894(0.012)	0.839(0.015)
	ResNet152	0.840(0.010)	0.685(0.017)	0.881(0.010)	0.849(0.014)	**0.921(0.012)**	0.891(0.013)
Category 3 Organ System	VGG16	0.655(0.015)	0.619(0.019)	0.689(0.014)	0.622(0.017)	0.691(0.014)	0.730(0.016)
	DenseNet121	0.667(0.013)	0.700(0.016)	0.691(0.013)	0.626(0.018)	0.690(0.013)	0.650(0.014)
	ResNet152	0.803(0.010)	0.674(0.018)	**0.854(0.012)**	0.795(0.014)	0.800(0.010)	0.790(0.015)
*AUC-PRC*							
Category 1 Modality	VGG16	0.322(0.019)	0.312(0.017)	0.379(0.017)	0.373(0.020)	0.590(0.016)	0.352(0.019)
	DenseNet121	0.287(0.021)	0.310(0.019)	0.407(0.016)	0.390(0.019)	0.572(0.018)	0.219(0.021)
	ResNet152	0.361(0.021)	0.208(0.018)	0.469(0.017)	0.343(0.019)	**0.618(0.016)**	0.224(0.018)
Category 2 Plane	VGG16	0.252(0.018)	0.368(0.018)	0.331(0.019)	0.370(0.021)	0.439(0.017)	0.288(0.020)
	DenseNet121	0.269(0.017)	0.279(0.021)	0.347(0.018)	0.335(0.021)	0.437(0.019)	0.351(0.019)
	ResNet152	0.248(0.020)	0.293(0.021)	0.365(0.017)	0.321(0.020)	**0.526(0.016)**	0.435(0.017)
Category 3 Organ System	VGG16	0.341(0.016)	0.348(0.020)	0.393(0.018)	0.289(0.019)	0.443(0.019)	0.351(0.016)
	DenseNet121	0.364(0.018)	0.420(0.018)	0.377(0.016)	0.289(0.021)	0.433(0.021)	0.330(0.018)
	ResNet152	0.428(0.017)	0.322(0.017)	0.473(0.019)	0.396(0.018)	**0.510(0.016)**	0.352(0.018)

#### Ablation study

To justify the importance of each component in *MedFuseNet*, we conducted an ablation study where we compare the performance of *MedFuseNet* against the various possible combinations of Image features, Question features, and Fusion techniques—for all the answer categorization task. We conduct ablation studies on 3 types of image features—VGG16, DenseNet121, and ResNet152; 2 types of question features—BERT and XLNet; and 3 types of fusion techniques - MCB, MUTAN, and MFB, along with the attention mechanisms. In total, there are 18 types of possible combinations that are tested and studied. The evaluation metric scores obtained for each possible combinations and for different question categories are summarized in Table 7. In terms of accuracy, *MedFuseNet* (BERT + ResNet + MFB) performs the best for question category 1 (Modality) with an accuracy of **0.840** and for category 2 (Plane) with an accuracy of **0.780**. Another close model for these two categories is BERT + DenseNet + MFB with 0.813 accuracy score for Modality and 0.757 for Plane. These scores suggest that image features are more generic for models with skip connections. Moreover, this asserts the power of MFB as a fusion model. For category 3 (Organ), the XLNet + ResNet + MFB combination achieves the best accuracy score of **0.844**.

In terms of AUC-ROC scores, BERT + VGG16 + MFB performs the best with a score of **0.954**, and is marginally ahead of our *MedFuseNet* with a score of 0.942 for Modality. For category 2 (Plane), our *MedFuseNet* again has the highest AUC-ROC score of **0.921**. Our *MedFuseNet* also performs well on category 3 questions with an AUC-ROC score of 0.800. The highest AUC-ROC score for category 3 is from BERT + ResNet + MUTAN with a value of **0.854**. These figures demonstrate that our *MedFuseNet* performs well with the inherent class imbalance in the data.

The trend for accuracy scores continues for AUC-PRC scores as well. *MedFuseNet* has the highest AUC-PRC for category 1 and category 2 with values of **0.618** and **0.526**, respectively. In category 3, the highest AUC-PRC is for BERT + XLNet + MFB with **0.578** followed by *MedFuseNet* with a score of **0.510**. This quantitative analysis establishes that our *MedFuseNet* is superior compared to all the other combinations with consistently performing and achieving the maximum scores for the majority of the metrics.

**Table 8:** Accuracy scores for the ablation study experiments of PathVQA yes-no answer type dataset.

Image Feature	MCB		MUTAN		MFB	
	BERT	XLNet	BERT	XLNet	BERT	XLNet
VGG16	0.614(0.014)	0.502(0.012)	0.637(0.014)	0.513(0.013)	**0.645(0.012)**	0.507(0.014)
DenseNet121	0.609(0.013)	0.503(0.014)	0.624(0.012)	0.514(0.013)	0.636(0.013)	0.507(0.012)
ResNet152	0.611(0.015)	0.505(0.014)	0.620(0.013)	0.505(0.012)	0.621(0.013)	0.503(0.015)

The results of a similar ablation study on the PathVQA yes-no type dataset is shown in Table 8. We observe that the combination of BERT + VGG16 + MFB performs best with an accuracy score of **0.645**. This is followed by BERT + VGG16 + MUTAN and BERT + DenseNet121 + MFB with accuracy scores of 0.637 and 0.636, respectively. The combination of BERT + ResNet152 + MFB has an accuracy score of 0.621. This ablation study again strengthens the claim that the PathVQA dataset for yes-no type answers is not very complex, which is also supported by the results of the baseline methods. Thus, simpler models like VGG16 and BERT tend to perform better for the answer categorization task for the PathVQA dataset.

**Table 9:**
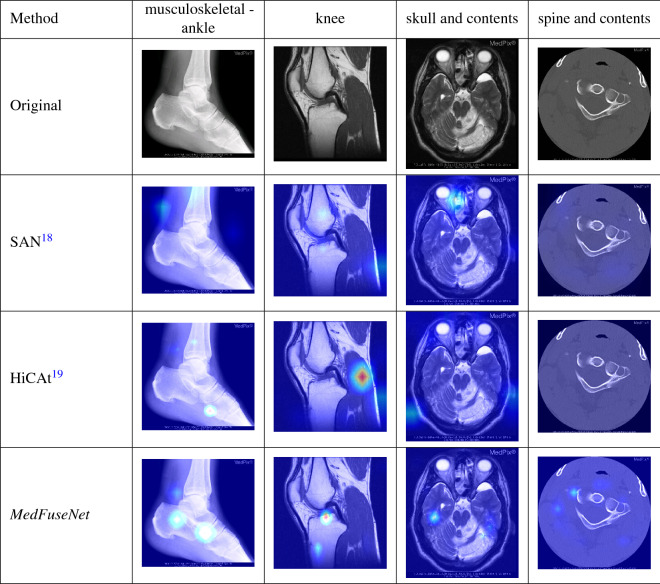
Image Attention visualization for SAN, Hie. Co-Att, and *MedFuseNet*.

### Attention visualization

Here, we perform the qualitative analysis of *MedFuseNet* and compare its results to the ones from SAN, and Hierarchical Co-Attention models. Since VIS+LSTM and Deeper-LSTM + Norm. CNN do not have any attention modules, we do not perform a qualitative analysis for these models. We visualized the image attention maps for each model to study and understand the performance of the model. These interpretable results are summarized in Table 9. We have considered four cases, where each image belongs to a different organ system. This helps us interpret how well the model is learning the underlying nuances of the medical images. As mentioned in the “Implementation details” section, we use two attention glimpses. For the first scan of the ankle, SAN can be seen to have a distributed attention span with a certain focus on the upper part of the ankle, while Hierarchical Co-Attention focuses on two different parts of the ankle. Our *MedFuseNet* has its attention maps spanned over the ankle joints and the lower bone. In the knee scan, SAN again fails to focus on the appropriate location in the image and has distributed attention. Hierarchical Co-Attention spans its attention to the posterior ligament. On the other hand, our *MedFuseNet* has a distributed attention span over the cartilage and the lower shin bone, also known as the tibia. These visualizations support the fact that *MedFuseNet* is able to attend to the crucial discriminatory parts of the organ. The third example case is a radiology scan of the skull. Our *MedFuseNet* again has attention maps catered to both halves of the skull. The fourth case we visualized is a CT scan of the spine and contents, and we see that from the attention maps of *MedFuseNet* is able to focus on different parts of the scan, thus justifying the prediction. Therefore, observing the visualization of the attention maps can provide us interesting interpretable insights on where the VQA models are focusing while trying to answer the questions related to the medical scans. Through the above qualitative analysis we have shown that our *MedFuseNet* is able to focus on the major distinguishing parts of the medical image which helps it to correctly answer questions in for the medical VQA tasks.

**Figure 5:**
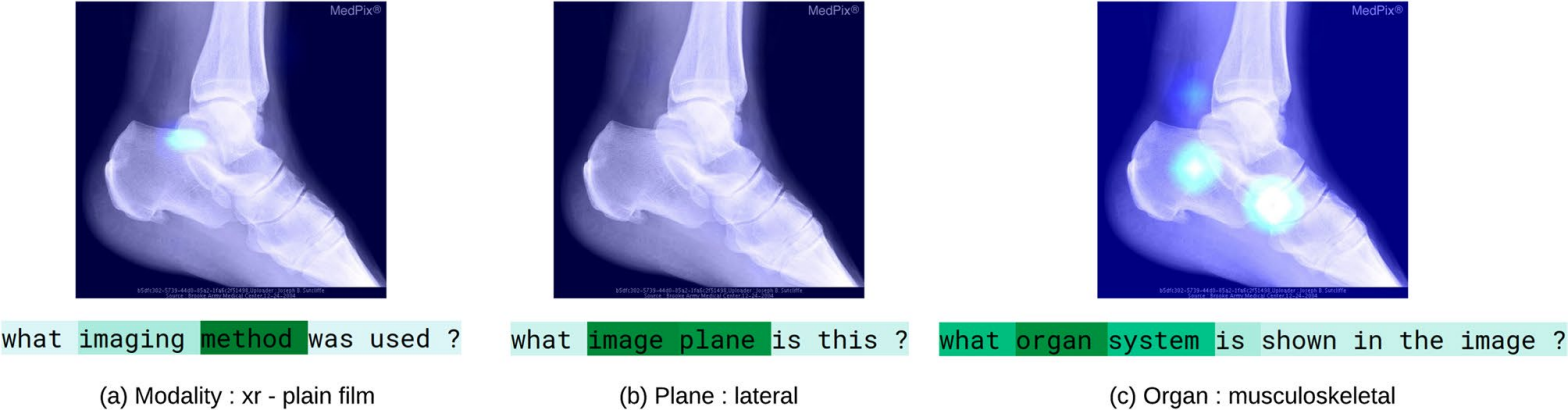
Co-Attention Maps for a sample case to display the attention span of *MedFuseNet* with the input image and the corresponding question attention. (**a**) Displays the image attention map and the corresponding question attention map for category 1—modality, (**b**) for category 2—plane, and (**c**) for category 3—organ.

In Fig. 5, we analyze the co-attention schema of the *MedFuseNet* model by laying the image and question attention maps for a particular case over the input image and question. For the first category, we can see that model spans its attention over keywords like “method” in the question which shows that the model is learning to be aware of the modality. Similarly, Fig. 5b shows how the model focuses on the keyword “plane” in the category 2 question. Through the image attention maps, we can infer that model has an evenly distributed attention to find the plane for the image. For category 3, again the question attention highlights the words like “organ” and “system”, thus, supporting the fact that the model knows where to span the textual attention. The image attention for category 3 also has a distributed attention span over multiple regions of the image.

**Figure 6:**
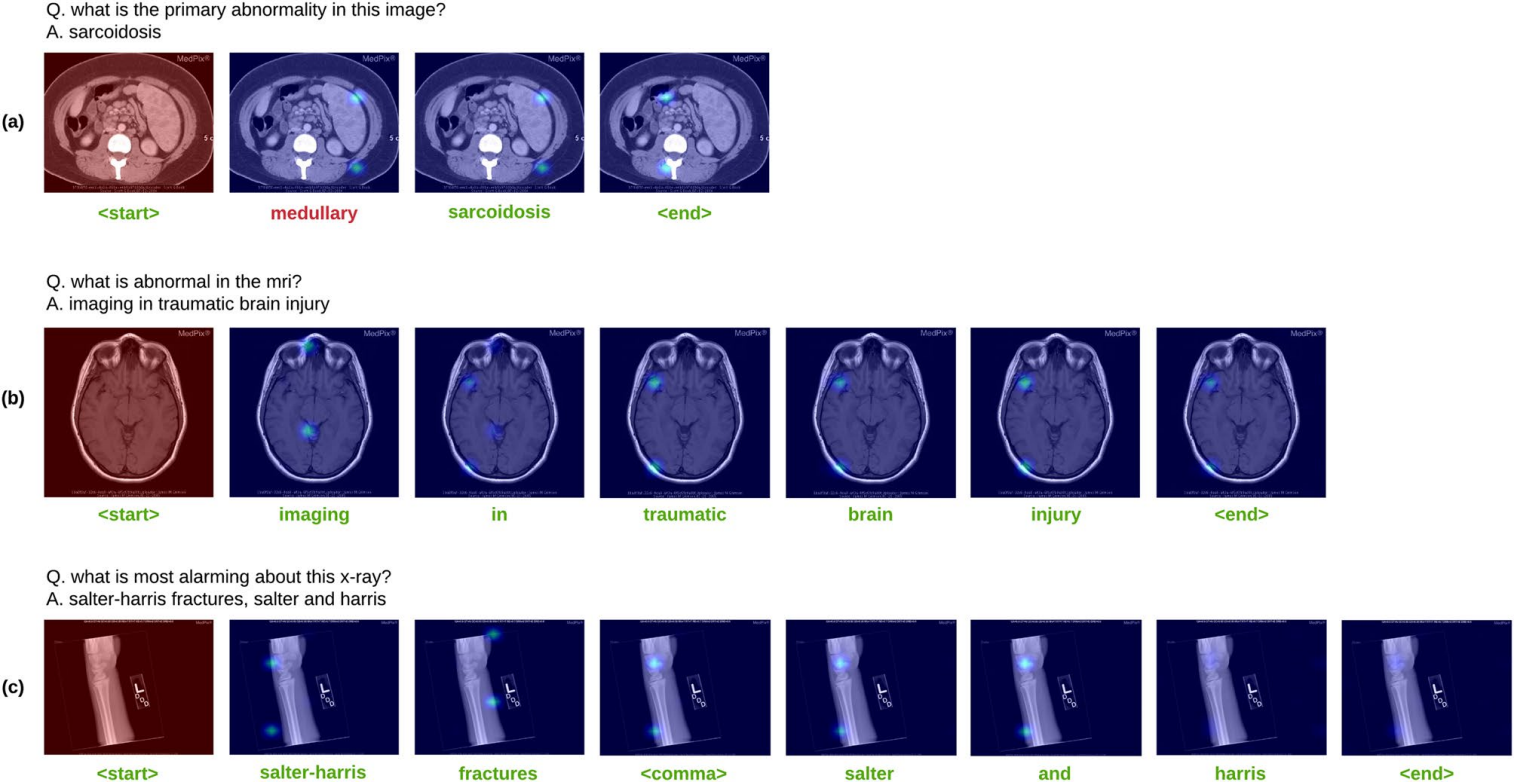
The attention maps produced by *MedFuseNet* while generating the words in the answer. There are three cases (**a**) sarcoidosis in the genitourinary system, (**b**) anoxic brain injury, and (**c**) salter-harris fracture in the bone.

In Fig. 6, we visualize the attention maps obtained from *MedFuseNet* while generating each word in the answer. As described in the “*MedFuseNet* model for medical VQA tasks” section, for each time step $t_i$, the attention maps of the previous time step $t_{i-1}$ are also fed into the LSTM. Figure 6 demonstrates the attention map that fed with each word to the model for three cases. The first case (a) is of sarcoidosis in the genitourinary organ system. Our *MedFuseNet* generates an extra word “medullary” which is related to the medulla oblongata, located in the stem of the spinal cord near the skull. For the other two cases, our model predicts the answer correctly along with the punctuation of comma (,). The second case (b) is of a brain injury. In this case, we can observe how our model is attending different parts of the brain to discover the cause of injury. The third case (c) is of salter and harris fracture, a fracture specifically caused at the joint of two bones. As we can see in the attention maps, our model is specifically attending at the joint portion of the scan multiple times while generating the words “salter-harris” and “salter”. This shows that the model is slowly and steadily learning to identify this special type of fracture and also localize it in the medical image. Thus, attention visualization of our *MedFuseNet* helps us to understand the model performance for the answer generation task.

## Conclusion

Visual questions answering systems for medical images can be extremely helpful in providing the doctors with a second-opinion. In this paper, we presented *MedFuseNet*—an attention-based multimodal deep learning model, for VQA on medical images. *MedFuseNet* is specifically tailored for handling medical images and it aims to learn the essential components of a medical image and effectively answer questions related to it. A rigorous quantitative and qualitative analysis of *MedFuseNet*’s performance was done on two real-world medical VQA datasets for two medical VQA tasks—answer categorization and answer generation tasks. Ablation study was conducted to investigate the role of image features, question features, and fusion techniques on the model performance for the two VQA tasks. For our future work, we will focus on improving and intergrating the decoder with our *MedFuseNet* for better answer generation task. We are also working on annotating a large VQA medical domain dataset for a diverse sets of scans, organs, and diseases.
